# Endogenous Synthesis of Corticosteroids in the Hippocampus

**DOI:** 10.1371/journal.pone.0021631

**Published:** 2011-07-28

**Authors:** Shimpei Higo, Yasushi Hojo, Hirotaka Ishii, Yoshimasa Komatsuzaki, Yuuki Ooishi, Gen Murakami, Hideo Mukai, Takeshi Yamazaki, Daiichiro Nakahara, Anna Barron, Tetsuya Kimoto, Suguru Kawato

**Affiliations:** 1 Department of Biophysics and Life Sciences, Graduate School of Arts and Sciences, The University of Tokyo, Meguro, Tokyo, Japan; 2 Core Research for Evolutional Science and Technology Project of Japan Science and Technology Agency, The University of Tokyo, Meguro, Tokyo, Japan; 3 Bioinformatics Project of Japan Science and Technology Agency, The University of Tokyo, Meguro, Tokyo, Japan; 4 Department of Physics, College of Science and Technology, Nihon University, Chiyoda, Tokyo, Japan; 5 Laboratory of Molecular Brain Science, Graduate School of Integrated Arts and Sciences, Hiroshima University, Higashi-Hiroshima, Japan; 6 Department of Psychology, Hamamatsu University School of Medicine, Hamamatsu, Japan; Biological Research Center of the Hungarian Academy of Sciences, Hungary

## Abstract

**Background:**

Brain synthesis of steroids including sex-steroids is attracting much attention. The endogenous synthesis of corticosteroids in the hippocampus, however, has been doubted because of the inability to detect deoxycorticosterone (DOC) synthase, cytochrome P450(c21).

**Methodology/Principal Findings:**

The expression of P450(c21) was demonstrated using mRNA analysis and immmunogold electron microscopic analysis in the adult male rat hippocampus. DOC production from progesterone (PROG) was demonstrated by metabolism analysis of ^3^H-steroids. All the enzymes required for corticosteroid synthesis including P450(c21), P450(2D4), P450(11β1) and 3β-hydroxysteroid dehydrogenase (3β-HSD) were localized in the hippocampal principal neurons as shown via *in situ* hybridization and immunoelectron microscopic analysis. Accurate corticosteroid concentrations in rat hippocampus were determined by liquid chromatography-tandem mass spectrometry. In adrenalectomized rats, net hippocampus-synthesized corticosterone (CORT) and DOC were determined to 6.9 and 5.8 nM, respectively. Enhanced spinogenesis was observed in the hippocampus following application of low nanomolar (10 nM) doses of CORT for 1 h.

**Conclusions/Significance:**

These results imply the complete pathway of corticosteroid synthesis of ‘pregnenolone →PROG→DOC→CORT’ in the hippocampal neurons. Both P450(c21) and P450(2D4) can catalyze conversion of PROG to DOC. The low nanomolar level of CORT synthesized in hippocampal neurons may play a role in modulation of synaptic plasticity, in contrast to the stress effects by micromolar CORT from adrenal glands.

## Introduction

The hippocampus is a target of corticosterone (CORT) modulatory actions, with high levels of glucocorticoid receptor (GR) and mineralocorticoid receptor (MR) expression [1], [2]. Traditionally, CORT had been thought to be synthesized exclusively in the adrenal cortex, reaching the brain via blood circulation. *De novo* synthesis of CORT from PROG in the brain has been doubted partly because brain CORT disappears after adrenalectomy (ADX) in rats [3]. However, recent evidence shows that the hippocampus expresses steroidogenic enzymes required for corticosteroid synthesis, including cytochromes P450scc, P450(11β1), P450(11β2), and 3β-hydroxysteroid dehydrogenase (3β-HSD) [4]-[10]. Some of these enzymes are also necessary for sex-steroid synthesis [11]-[14]. Yet, the complete corticosteroid synthesis of ‘pregnenolone (PREG) → progesterone (PROG)→ deoxycorticosterone (DOC)→CORT or aldosterone (ALDO)’ in the hippocampus has not been proven (see Fig. S1). Although previous studies have shown parts of the corticosteroid synthesis pathway in brain, including, PREG→PROG and DOC→CORT and DOC→ALDO[5], [6], [10], the conversion of PROG→DOC to be demonstrated. Cytochrome P450(c21) (DOC synthase), a key enzyme catalyzing the conversion of PROG to DOC, has not been detected in the hippocampus, although mRNA expression has been demonstrated in other brain regions including the hypothalamus, cortex, cerebellum and striatum [15], [16].

In the current study, we demonstrated expression and activity of P450(c21) and the complete metabolism of PROG→DOC→CORT in rat hippocampus. In addition to P450(c21), expression of cytochrome P450(2D4), another candidate for DOC synthase [16], [17], was also observed. Further, the net hippocampus-derived CORT content was determined in ADX rats by mass-spectrometric analysis. We further investigated the effect of low dose CORT on spinogenesis of hippocampal neurons in order to show a possible role of hippocampus-derived CORT.

## Results

### Molecular biological analysis of corticosteroid synthesizing enzymes

**Figure 1:**
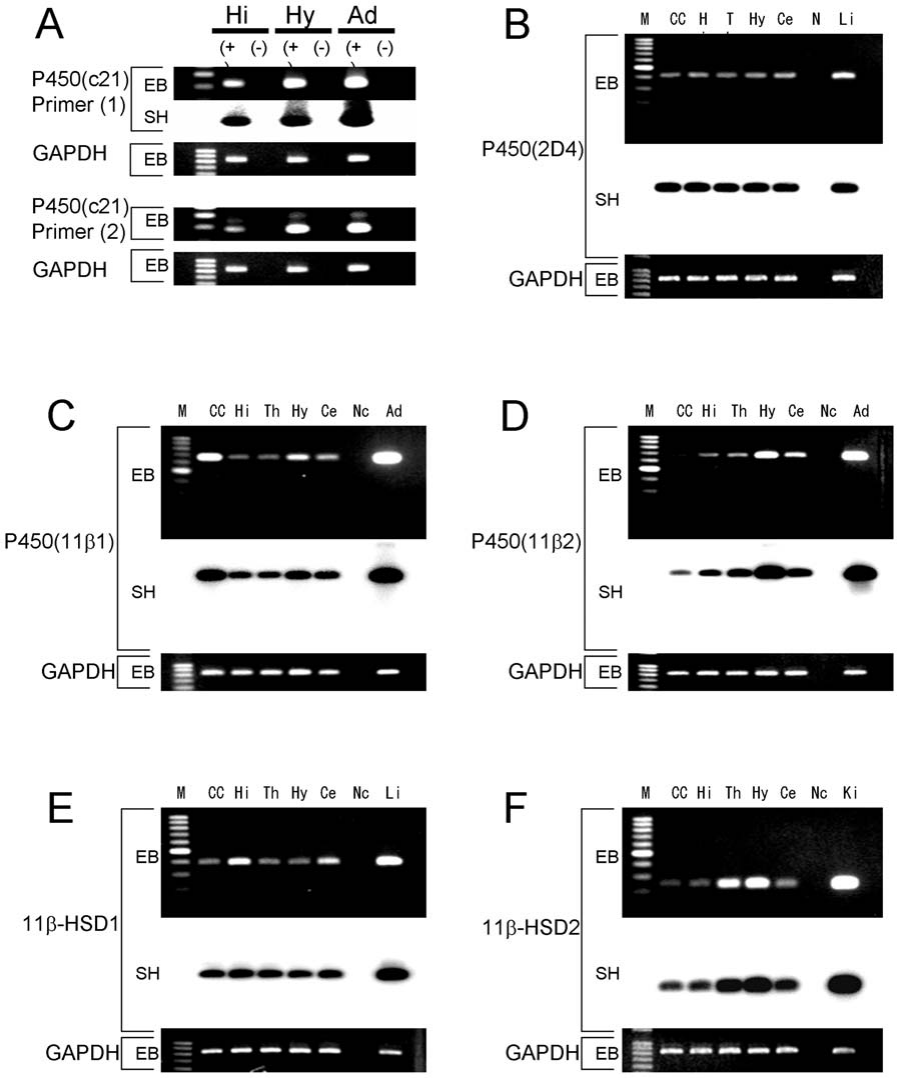
RT-PCR analysis of enzymes required for corticosteroid synthesis in the adult (12-week old) male brain. (A) P450(c21), (B) P450(2D4), (C) P450(11β1), (D) P450(11β2), (E) 11β-HSD (type 1), and (F) 11β-HSD (type 2). In panel A, from left to right, size marker (100 bp ladder), Hi(+) hippocampus with template DNA, Hi(-) hippocampus without template DNA, Hy (+) hypothalamus with template DNA, and Hy (-) hypothalamus without template DNA. The right most is the adrenal (Ad (+)) as the positive control. In P450(c21), the primer pairs (1) (top) identifies only an active form of P450(c21). Both active form (281 bp) and inactive form (351 bp) are observed with primer pairs (2) (bottom). By comparison, almost all of hippocampal P450(c21) is shown to be an active form.In each panel from left to right (B–F), size marker (M), cortex (CC), hippocampus (Hi), thalamus (Th), hypothalamus (Hy), cerebellum (Ce), the sample without template DNA as negative control (Nc), and positive control. Liver (Li) for (B) and (E), adrenal (Ad) for (C) and (D), and kidney (Ki) for (F) are used as positive control samples. For each enzyme, the RT-PCR products for mRNAs are visualized with ethidium bromide staining (EB) on the top of each panel. Southern hybridization (SH) of cDNA is also shown on the middle of each panel. As an internal control, the ethidium bromide staining of glyceraldehydes-3-phosphate dehydrogenase (GAPDH) is shown on the bottom of each panel. Total RNAs used are 50 ng for each enzyme.

Cellular localization and expression of enzymes responsible for CORT synthesis including P450(c21), P450(2D4), P450(11β1), P450(11β2), and 11β-HSD (types 1 and 2) were examined. Typical RT-PCR patterns of mRNA transcripts are shown in Fig. 1. Because the expression level of P450(c21) is extremely low in the hippocampus, we performed careful primer design using minimization of Gibbs free energy (ΔG) upon hybridization of primers with target sequences. We also choose primers with ΔG for 5 bases in 3′-side of the primer to be larger than average ΔG for improved specificity, because the specificity of primer-target recognition is mainly governed by 3′-side sequence of the primer. In the current study, ΔG for 5 bases in 3′-side of the P450(c21) primer was –6 kcal/mol, which was larger than the average ΔG for the P450(c21) primer ( = <1?show=[to]?>–8 kcal/mol) (Fig. S2). As a result, we succeeded the detection of P450(c21) mRNA.

Relative number of transcripts, expressed in the hippocampus of adult male rats, was in the order of 1/20,000 of that in the adrenal gland for P450(c21), almost same level of that in the liver for P450(2D4), in the order of 1/10,000 of that in the adrenal gland for P450(11β1), in the order of 1/5,000 of that in the adrenal gland for P450(11β2), approximately 1/50 of that in the liver for 11β-HSD (type 1), and approximately 1/500 of that in the kidney for 11β-HSD (type 2) (Fig. 1). The mRNA expression levels of P450(c21), P450(11β1), P450(11β2) and 11β-HSD (type 2) in the hippocampus were lower than those in the hypothalamus. The expression level of P450(2D4) in the hippocampus was almost equal of that in the hypothalamus. The expression level of 11β-HSD (type 2) was higher than that in the hypothalamus.

**Figure 2:**
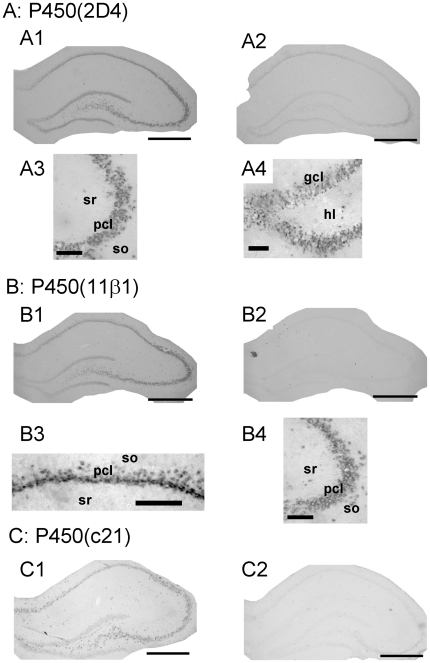
*In situ* hybridization analysis of P450(2D4) (A), P450(11β1) (B) and P450(c21) (C). Reaction time for color development is 12 h. (A1–2) Coronal sections of the whole hippocampus hybridized with antisense (A1) or sense (A2) probes for P450(2D4). (A3–4) The magnified images of CA3 (A3) and DG (A4) in the hippocampus hybridized with antisense probes for P450(2D4). (B1–2) Coronal sections of the whole hippocampus hybridized with antisense (B1) or sense (B2) probes for P450(11β1). (B3–4) The magnified images of CA1 (B3) and CA3 (B4) in the hippocampus hybridized with antisense probes for P450(11β1). (C1–2) Coronal sections of the whole hippocampus hybridized with antisense (C1) or sense (C2) probes for P450(c21). P450(2D4), P450(11β1) and P450(c21) are expressed in pyramidal neurons in CA1–CA3 region and granule cells in DG. Their expression in glial cells is weak. Scale bar, 800 µm for (A1–2), (B1–2) and (C1–2); 120 µm for (A3), (A4), (B3) and (B4). Abbreviations: gcl, granule cell layer; hl, hilus; pcl, pyramidal cell layer; so, stratum oriens; sr, stratum radiatum.

The cellular localization of P450(2D4), P450(11β1) and P450(c21) was identified using *in situ* hybridization and immunohistochemical staining. Significant expressions of both P450(2D4) and P450(11β1) were observed in pyramidal neurons (CA1, CA3) and granule neurons (DG) and these enzymes were weakly expressed in glial cells (Fig. 2 and S3). The weak expression of P450(c21) was observed in pyramidal and granule neurons (Fig. 2C). Because the expression level of mRNA for P450(c21) was very low, the Tyramide signal amplification system was used to obtain sufficient sensitivity.

### Cellular and Subcellular localization of enzymes for corticosteroid synthesis

**Figure 3:**
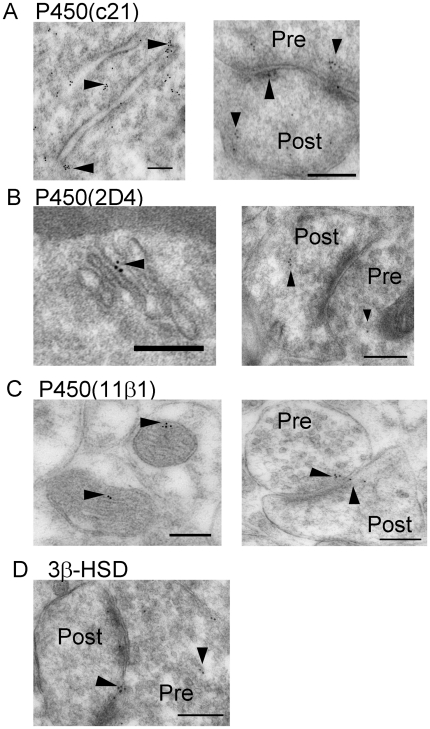
Immunoelectron microscopic analysis of the distribution of the enzymes required for corticosteroid synthesis within the axospinous synapses, in the stratum radiatum of the hippocampal CA1 region. (A) P450(c21), (B) P450(2D4), (C) P450(11β1), and (D) 3β -HSD (type 1). P450(c21) and P450(2D4) are localized not only in the endoplasmic reticulum but also in both the axon terminals and dendritic spines of principal neurons (A, B, arrowheads). P450(11β1) is localized in both the mitochondria and synapses of the principal neurons (C, arrowheads). post, postsynaptic region (spine head). Scale bar, 200 nm, except for (B) left (100 nm).

Immunoelectron microscopic analysis using postembedding immunogold was performed in order to determine the localization of enzymes for corticosteroid synthesis (P450(c21), P450(2D4), P450(11β1) and 3β-HSD) in the hippocampus. This method is particularly useful to detect enzymes with extremely low expression level such as P450(c21). All enzymes were mainly localized in principal neurons including pyramidal neurons of CA1 and CA3 regions as well as granule neurons of the DG. Weak expression was observed in some glial cells. P450(c21) and P450(2D4) were localized not only in the endoplasmic reticulum but also in both the axon terminals and dendritic spines of principal neurons (Fig. 3A, 3B). P450(11β1) was localized in both the mitochondria and synapses of principal neurons (Fig. 3C). We also observed 3β-HSD in the synapses in addition to the endoplasmic reticulum (Fig. 3D).

The subcellular localization of these enzymes was confirmed by western blot with purified fractions of postsynaptic density, endoplasmic reticulum and mitochondria (see Fig. S4 and Text S1).

### Mass-spectrometric analysis of corticosteroids in the hippocampus

**Figure 4:**
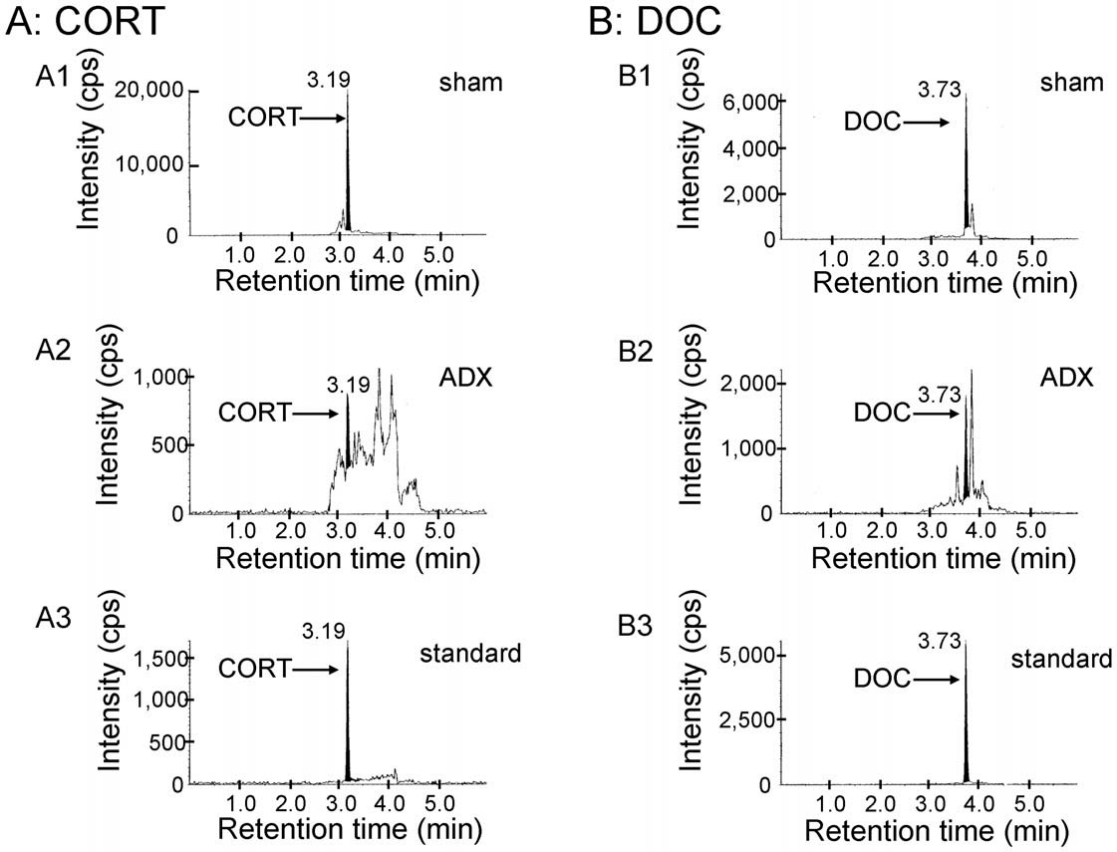
Mass-spectrometric analysis of hippocampal corticosteroids. LC-MS/MS ion chromatograms of CORT (A) and DOC (B). (A1), and (B1) represent the chromatograms of the fragmented ions of each steroid from the hippocampus of ADX rats. Shaded portions indicate the intensity of fragmented ions of CORT (m/z = 121), and DOC (m/z = 97), respectively. (A2) and (B2) represent the chromatograms of the fragmented ions of the standard steroids. The vertical axis indicates the intensity of the fragmented ion. The horizontal axis indicates the retention time of the fragmented ion, t = 3.19 min for CORT (A1), and t = 3.73 min for DOC (B1). The time of the injection to LC system is defined as t = 0 min. Note that pre-purification step using normal phase HPLC before injection to LC system is very important to achieve high precision and good reproducibility of LC-MS/MS determination in order to avoid contamination of other steroids and fats. Steroids are further separated with reversed phase LC-column. In the multiple reaction monitoring mode, the instrument monitored the m/z transition (Table S2).

The concentration of CORT and DOC was determined in steroid extracts from adult male rat hippocampus using a chromatogram analysis of the fragmented ions (Fig. 4). After selection of mother ions, fragmentation and detection were performed via MS/MS procedures. Chromatographic profiles for the fragmented ions of CORT (m/z = 121) showed a clear peak with the retention time of 3.19 min which was the same as that of the fragmented ion of the standard CORT, indicating a good specificity of the analysis (Fig. 4A). In the chromatographic profiles of the fragmented ion of DOC (m/z = 97), a single peak was observed at 3.73 min (Fig. 4B).

In order to determine the net corticosteroids synthesis in the hippocampus, we used ADX rats to eliminate adrenal-derived steroids (CORT and DOC) via the blood circulation. Results are summarized in Table 1. Two weeks after ADX, the concentrations of CORT in the hippocampus and plasma were 2.4 ng/g wet weight ( = 6.9 nM) and 0.8 ng/ml (2.3 nM), respectively. The DOC concentration was 1.9 ng/g wet weight (5.9 nM) in the hippocampus, and 0.5 ng/ml (1.4 nM) in plasma, respectively.

**Table 1:** Mass spectrometric analysis of the concentration of steroids in the hippocampus and plasma of adult ADX male rats.

	Hippocampus	Plasma
CORT (ng/g wet weight or mL)	2.4±0.6^a^ (n^b^ = 11)	0.8±0.3 (n = 11)
CORT (nM)	6.9±1.8	2.3±0.9
DOC (ng/g wet weight or mL)	1.9±0.5 (n = 23)	0.5±0.1 (n = 23)
DOC (nM)	5.8±1.4	1.4±0.3

a Data are expressed as mean ± SEM.

b Number of animals (i.e. the number of hippocampi).

Even at 4 weeks after the ADX, the level of CORT did not decrease further from the level at two weeks within experimental error (±2.1 nM, SEM). These results imply that the circulation-derived CORT in the hippocampus was cleaned up already at two weeks after ADX, therefore 6.9 nM is solely hippocampus-synthesized CORT.

### Validation of mass-spectrometric analysis

To confirm the assay accuracy, the hippocampal homogenate spiked with known amounts of the steroids was prepared and its concentration of steroid was determined (Table S1). Satisfactory accuracy was obtained, supporting the accuracy of determined hippocampal steroid content in Table 1. The limits of quantifications (LOQs) were defined in Table S2 as the lowest value with an acceptable accuracy (91.6–107.1 %) and precision (i.e. RSD<11 %). The results of intra- and inter-assay were shown in Table S2. The RSD for intra- and inter-assay was less than 10.2 % and 10.9 %, respectively. These results indicate that the present method is highly reproducible and accurate.

In addition, we observed approximately 0.68 nM of 11-deoxycortisol (roughly 1/10 of DOC) in the hippocampus. Because P450(17α) is expressed in the hippocampus, DOC→11-deoxycortisol → cortisol pathway may also work.

### Hippocampal corticosteroid metabolism

**Figure 5:**
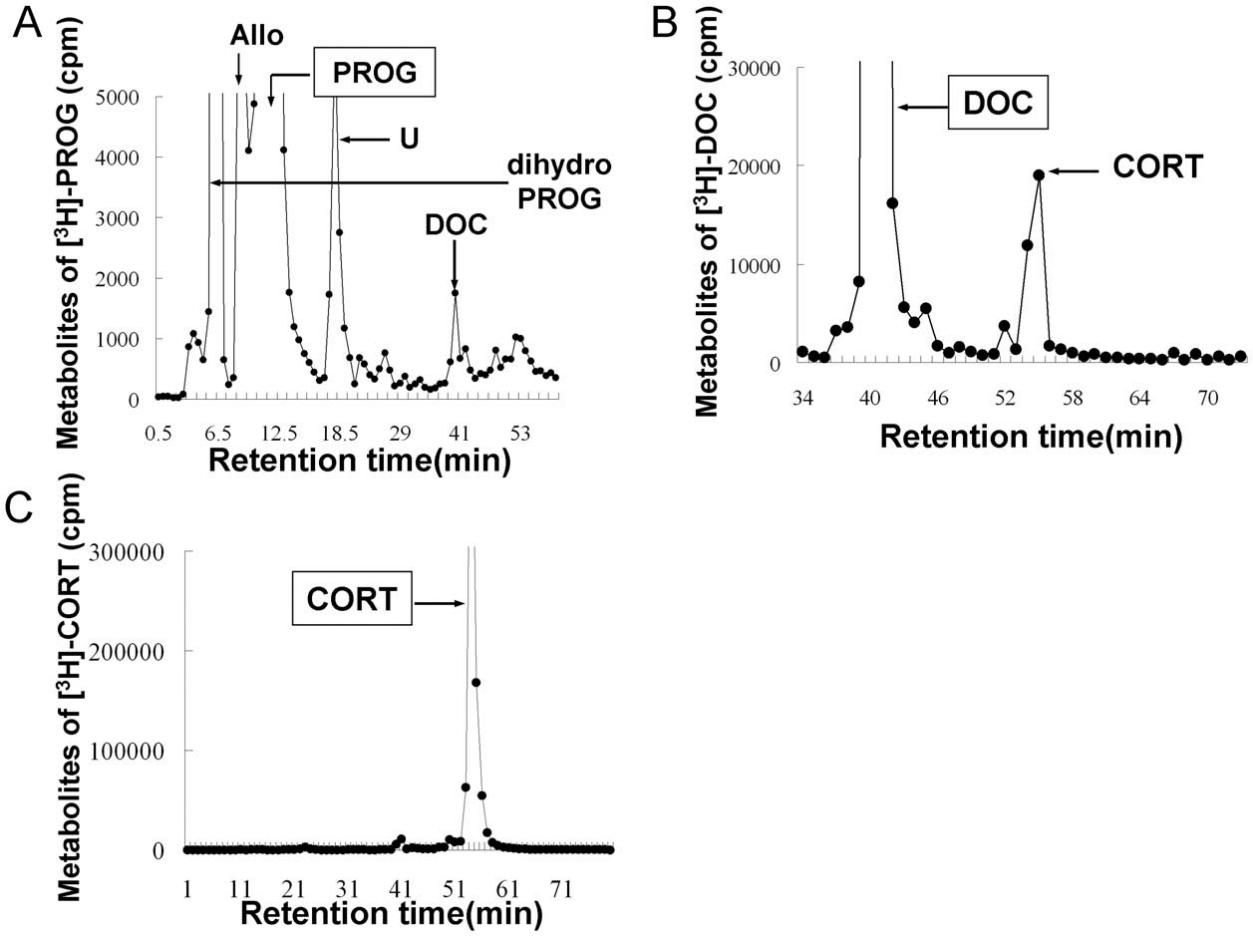
Normal phase HPLC analysis of steroid metabolism in the hippocampal slices from adult male rat. (A) Typical HPLC profiles of ^3^H-PROG metabolites. DOC, allopregnanolone (Allo), dihydroprogesterone (dihydro PROG) are produced. U is unknown product. As previously reported [42], [43], 5α-DHPROG and allopregnanolone are observed. Several unknown peaks are also observed in the metabolism analysis. These unknown products may be 7-hydroxylated steroids (such as 7α-OH PROG) or 20-hydroxylated steroids, because the expression of Cyp7b and 20α-hydroxylase in the rodent hippocampus are reported [44], [45]. (B) Typical HPLC profiles of ^3^H-DOC metabolites. (C) Typical HPLC profiles of ^3^H-CORT metabolites. The incubation buffer contains 100 nM of finasteride, a specific inhibitor of 5α-reductase, in order to suppress the synthetic pathway of 5α-reduced metabolites such as allopregnanolone, because 5α-reductase is expressed in hippocampal principal neurons [18]. A portion of the purified radioactive metabolites (total of 10^6^ cpm) is analyzed using an HPLC system. Closed rectangle indicates the steroid used as substrate. Others indicate products from substrate. The elution peak positions (arrows) are calibrated with standard ^14^C-steroids. The retention time of the (same) standard ^14^C-steroid differed between each panel, due to the different elution experiments using the different silica gel columns. The vertical axis indicates ^3^H radioactivity (cpm). Three to five independent experiments with different animals were performed for each of these analyses, showing good reproducibility.

Analysis of the pathway of corticosteroid metabolism is necessary (Fig. S1), because mass-spectrometric determination shows only the contents of individual steroids. The metabolism of radioactive steroids in hippocampal slices was investigated using normal phase HPLC. Hippocampal slices were incubated with 5×10^6^ cpm of ^3^H-labeled steroid substrate for 5 h. Typical results of HPLC analysis are illustrated in Fig. 5. A significant production of DOC from ^3^H-PROG was observed (Fig. 5A). The incubation buffer contains 100 nM of finasteride, a specific inhibitor of 5α-reductase, in order to reduce the conversion to 5α-reduced metabolites such as allopregnanolone. Because of the strong activity of 5α-reductase and 3α-HSD [18], some 5α-dihydro PROG and TH-PROG (allopregnanolone) were produced even in the presence of finasteride. In the absence of finasteride, the production of DOC and CORT were very weak to observe. When ^3^H-DOC was used as a substrate, CORT from DOC was produced (Fig. 5B). The conversion of CORT to other steroids was very weak (Fig. 5C), indicating that CORT is stably present once it produced.

### Low dose effect of CORT on spinogenesis of hippocampal neurons

**Figure 6:**
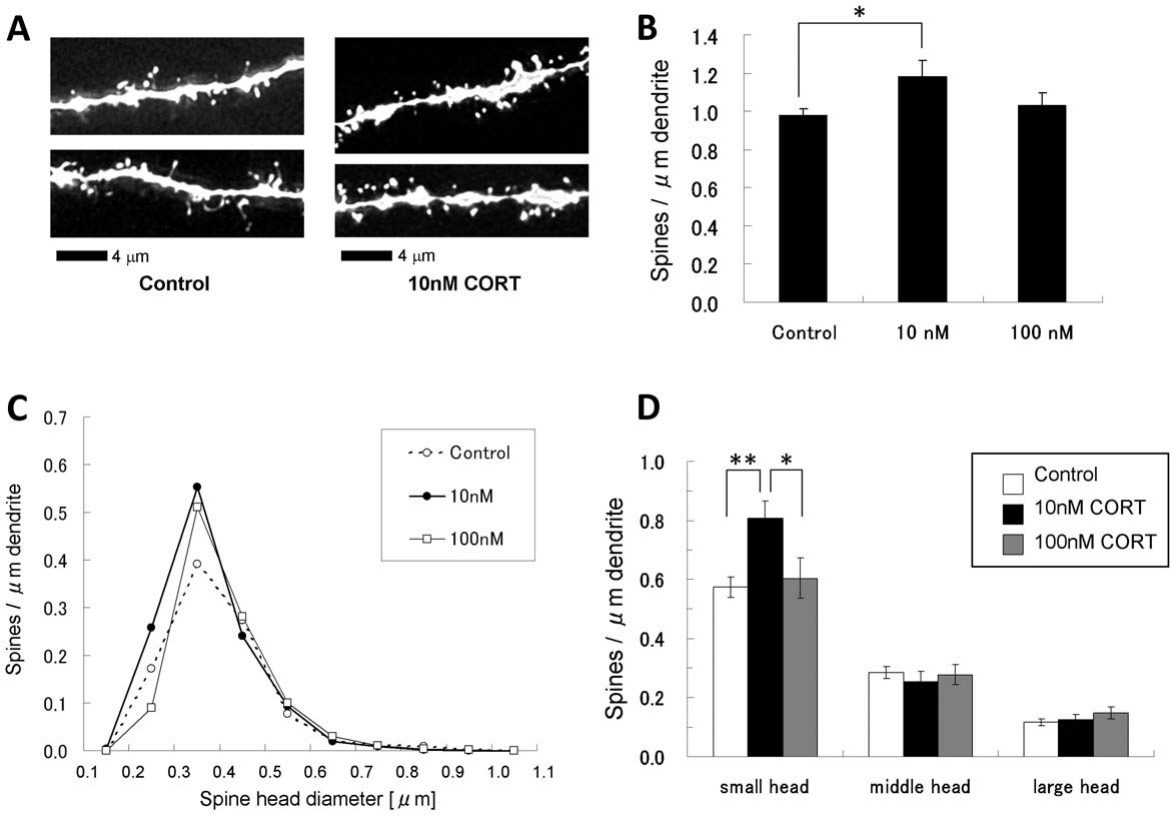
Changes in the density and morphology of spines by low dose CORT in hippocampal slices. Spines were analyzed along the secondary dendrites of pyramidal neurons in the stratum radiatum of CA1 neurons. Small-, middle-, and large-head spines are different in the density of α-amino-3-hydroxy-5-methyl-4-isoxazolepropionic acid (AMPA) receptors [46], therefore these three types of spines may have different capacity of synaptic transmission. The density of AMPA receptors in the spine is roughly proportional to its postsynaptic density (PSD) area size, whereas the density of NMDA receptors is inversely proportional to the PSD area size. (A) Maximal intensity projections onto XY plane from z-series confocal micrographs, showing spines along the secondary dendrites of hippocampal CA1 pyramidal neurons. Dendrites without CORT-treatments (Control) and with 10 nM CORT treatment for 1 h (10 nM CORT). Bar, 4 µm. (B) Low dose CORT treatments on the total spine density. A 1 h treatment in ACSF without drugs (0 nM), with 10 nM CORT (10 nM), with 100 nM CORT (100 nM). Vertical axis is the average number of spines per 1 µm of dendrite. (C) Histogram of spine head diameters. After a 1 h treatment in ACSF without CORT (Control, open circle), with 10 nM CORT (10 nM, closed black circle), and with 100 nM CORT (100 nM, open square). Vertical axis is the average number of spines per 1 µm of dendrite. (D) Density of three subtypes of spines. From left to right, small-head spines (small), middle-head spines (middle), and large-head spines (large) are categolized. ACSF without CORT (Control, open column), 10 nM CORT (10 nM, black column), and 100 nM CORT (100 nM, hatched column) are shown. In (B) and (D), results are reported as mean ± SEM. For each CORT treatment, we investigated, 3 rats, 6 slices, 12 neurons, 24 dendrites and roughly 1200 spines. In (B) and (D), the significance of CORT was examined using the Tukey–Kramer *post hoc* multiple comparisons test when one way ANOVA tests yielded *P*<0.05. *P<0.05, **P<0.01.

To determine the potential physiological significance of nanomolar concentrations of hippocampal CORT, we investigated CORT effects on dendritic spine density and morphology in hippocampal ‘acute’ slices. It should be noted that CORT levels were depleted in control ‘acute’ slices, containing only 1.9 nM CORT following 2 h recovery incubation in ACSF. Treatment with exogenous 10 nM CORT for 1 h significantly increased the total spine density (1.18 spines/µm) compared with control slices (0.98 spines/µm, with no exogenous CORT) (Fig. 6).On the other hand, treatment with 100 nM CORT only slightly increased the total spine density.

Morphological changes in spine head diameter induced by 1 h CORT administration were also assessed. Spines were classified into three categories based on head diameters: small-head spines (0.2–0.4 µm): middle-head spines (0.4–0.5 µm): and large-head spines (0.5–1.0 µm). Morphological categorization of spines into three subclasses enabled complex responses in spine subpopulations upon CORT application to be distinguished. Treatment with 10 nM CORT significantly increased the density of small-head spines from 0.58 (control) to 0.81 spines/µm, while the density of middle-head spines (approx. 0.28 spines/µm) and large-head spines (approx. 0.12 spines/µm) was not significantly altered (Fig. 6). Morphological changes induced by 100 nM CORT were smaller than those induced by 10 nM CORT. The majority of spines (>95%) had a distinct head and neck, therefore these analysis cover major populations of spines.

## Discussion

In the current study, we clarified the complete pathway for corticosteroid synthesis ‘PREG→PROG→DOC→CORT’ in hippocampal neurons. We demonstrated for the first time the expression, neuronal localization and activity of P450(c21) in the hippocampus. In addition, the localization of P450(2D4), another enzyme participating in DOC synthesis, was demonstrated.

### Previous investigations of brain corticosteroids

#### PROG→DOC conversion and P450(c21) expression

To date, direct evidence of DOC synthesis in the adult brain had not been reported. No expression of P450(c21) in the rat/mouse hippocampus has been reported, although a few studies have reported weak expression of P450(c21) mRNA in some regions of the rat brain such as hypothalamus, striatum, cerebellum [15], [16], [19]. As an exception, weak expression of P450(c21) mRNA was observed in the human hippocampus, with levels 1/20,000 of that of the adrenal gland [20]. Thus far, it has been believed that P450(c21) is not physiologically active in the hippocampus, and all CORT in the hippocampus is derived from the adrenal glands via the blood circulation. Although P450(2D4) has been known as the drug metabolizing P450, recently, it has been demonstrated that P450(2D4) can also convert PROG to DOC in hippocampus [16]. Therefore, both P450(c21) and P450(2D4) could contribute to DOC production. We observed the evidence of conversion of PROG to DOC, confirming de novo synthesis of DOC.

#### DOC→CORT conversion and P450(11β1) expression

The expression of P450(11β1) in the hippocampus has been described in several studies and neuronal localization of P450(11β1) has been demonstrated using immunohistochemistry [6], [10], [21]. We observed the localization of P450(11β1) in cell bodies of pyramidal and granule neurons at mRNA and protein level. Gomez-Sanchez and co-workers observe a weak CORT production from ^3^H-DOC, roughly 0.4 pmol/mg/3 h in the hippocampus [6]. However, they doubted *de novo* synthesis of CORT from PROG since CORT levels in whole brain were nigligible after adrenalectomy (ADX) of rats [3]. According to their report, CORT in the whole brain of five ADX rats was below the detection limit, but four ADX rats had measurable CORT in the brain (0.6 ng/g = 1.8 nM). Therefore, their results do not completely eliminate a possibility of the presence of brain-synthesized CORT. It should be noted that the expression level of P450(11β1) in the hippocampus does not change upon ADX [21]. In the hippocampus of ADX rats we observed the direct conversion of DOC to CORT (Fig. 5) as well as low, but physiologically significant level of CORT. The expression of P450(11β1) (both mRNA and protein) in neurons supports endogenous synthesis. Interestingly, the expression level of P450(11β1) is roughly 10-fold more in the cortex than in the hippocampus.

### Functional significance of hippocampus-synthesized corticosteroids

The low nanomolar level of CORT, synthesized in the hippocampal neurons, may play a essential role in enhancement of synaptic plasticity, in contrast to the deleterious effects (e.g., neuronal cell death or shrinkage of dendrites) elicited by micromolar plasma CORT secreted from the adrenal gland under stressful conditions [22], [23]. In the current study, low dose CORT (10 nM) enhanced spinogenesis, particularly increasing the density of small-head spines (Fig. 6). It has been previously demonstrated that nanomolar doses of CORT (10 nM) drives the Erk MAP kinase pathway, increasing both the expression and phosphorylation of MAP kinase [24]. Taken together, hippocampus-synthesized CORT (approx. 7 nM) might induce spinogenesis via activation of the Erk MAP kinase pathway.

Earlier studies have also shown a range of beneficial effects of nanomolar doses of CORT including strengthened signaling, neuroprotection as well as a potential role in development [25], [26]. In vivo, low plasma levels of CORT increased the amplitude of population spike, as did application of low concentration (5–50 nM) of CORT to CORT-depleted slices [26]. Low doses of CORT may also have neuroprotective effects. For example, low dose CORT attenuates dexamethasone – induced apoptosis in dentate gyrus via MR signaling pathway [27]. Nanomolar low dose of CORT may also play a role in neuronal development, with 5–10 nM CORT found to enhance differentiation of neural stem cells by inducing the expression of astrocyte marker GFAP (J Kanno, K Igarashi, K Tanemura, H Asano, K Nakashima., 14^th^ Int Cong of Endocrinol, Kyoto, 2010, P8-3-1).

Importantly, all the 7 nM of hippocampal CORT could participate in modulation of neuronal functions such as spinogenesis, because corticosteroid binding globulin (CBG) and serum albumin are not present in the hippocampus, therefore all the hippocampal synthesized CORT should be biologically active. The persistence of a significant concentration of corticosterone in the plasma (2 nM) even after ADX may be derived from local synthesis in other peripheral tissue such as adipocytes. Although we have a significant concentration of CORT in the plasma, the majority (95–98%) of plasma CORT may be biologically inactive in basal conditions, with the majority of plasma CORT (90%) bound to CBG and a large proportion of the remaining CORT bound to serum albumin [28]. Although basal plasma CORT levels range between 100 and 300 nM, biologically active free CORT comprises only 2–5% of the total plasma CORT levels [29].

### Moderate CORT level is kept by weak CORT production in the hippocampus

A trace amount of endogenous DOC and CORT had been very difficult to detect by ^3^H-steroid metabolism analysis, because many different pathways exist other than PROG→DOC→CORT. Therefore, we employed mass-spectrometric assay for detection of a trace amount of these steroids in the hippocampus of ADX rats. We determined the concentrations of DOC and CORT to be 5.8 nM and 6.9 nM, respectively. These values are comparable to hippocampus-synthesized sex steroids such as estradiol (roughly 7 nM), testosterone (3 nM) and dihydrotestosterone (0.6 nM) [18].

Because the volume of hippocampus is very small (nearly 0.1 mL for one whole hippocampus), the calculated concentrations were relatively high in nM range for DOC and CORT. The absolute contents of DOC and CORT were only around 0.27 ng and 0.34 ng in one hippocampus with a weight of 0.14 g, respectively (Table 1). Although steroid production capacity is strong in the adrenal gland, circulation levels of steroids are diluted due to approx. 20 mL of blood (200-fold of the hippocampal volume). Though the hippocampal expression levels of enzymes (such as P450(c21) and P450(11β1)) are roughly 1/20,000 and 1/10,000 of those in the adrenal, they need to fill only small hippocampal volume (1/200 of the blood volume).

It should be noted that the hippocampus-synthesized CORT is stably present for even 5 h once produced (Fig. 5C), resulting in keeping the CORT level sufficient for modulation of synaptic plasticity. It may be due to roughly 1000-fold higher expression level of 11β-HSD (type 1) in the hippocampus than that of 11β-HSD (type 2) as judged from the cycle number of PCR (Figs. 1E and 1F). Because 11β-HSD (type 1) converts 11-dehydrocorticosterone (inactive form) to CORT, hippocampal CORT is prevented from inactivating once produced (Figs. 5C). On the other hand, in peripheral tissues such as kidney, the expression level of 11β-HSD (type2) is higher than that of 11β-HSD (type1).

### Role of MR and GR

In ADX rats, the stress due to application of ether anesthetics before decapitation did not increase hippocampal CORT level to a high stress-level (e.g., 0.5–1 µM), therefore hippocampus-synthesized CORT (roughly 7 nM) may not play a role in the negative feedback by occupying many GR. Under basal conditions, all MR may be occupied by ALDO or CORT. Negative feedback would be induced by adrenal CORT. In response to stress, 0.5–1 µM adrenal CORT, a part of which penetrates into the hippocampus, could further occupy many unoccupied GR in the hippocampus and could replace ALDO on MR.

### Conclusion

The hippocampus is equipped with not only sex-steroid synthesis systems [11], [13], [14], [30] but also corticosteroid synthesis systems. Synaptic localization of important enzymes including P450(c21), P450(2D4) and P450(11β1) suggests a potential synaptocrine function of corticosteroids in hippocampal neurons. These enzymes are also localized in the endoplasmic reticulum (microsome) and mitochondria of hippocampal neurons, suggesting potential synaptocrine function of corticosteroid synthesis in hippocampal neurons. The current study opens new field of investigations concerning possible physiological function of hippocampus-synthesized nanomolar CORT, such as modulation of synaptic plasticity.

## Materials and Methods

### Animals

Young adult male Wistar rats (12-week old) were purchased from Saitama Experimental Animals Supply (Japan). All animals were maintained under a 12 h light/12 dark exposure and free access to food and water. Adrenalectomy (ADX) and sham operations were performed two-weeks before the experiments. The experimental procedure of this research was approved by the Committee for Animal Research of the University of Tokyo (The permission number is 19–10.).

### Chemicals

Corticosterone (CORT), deoxycorticosterone (DOC) and progesterone (PROG) were purchased from Sigma (USA). [−^13^C_3_]PROG was from Hayashi Pure Chemical (Japan). CORT-d_8_ and DOC-d_8_ were from CDN Isotope Inc. (Canada). Finasteride was from Aska Pharma Medical (Japan). [^3^H] or [^14^C] labeled steroids were purchased from Perkin Elmer (USA) and their specific activities were 76.5 Ci/mmol ([1,2,6,7-^3^H]-CORT) and 102 Ci/mmol ([1,2,6,7-^3^H]-PROG). [1,2-^3^H]-DOC (50 Ci/mmol) was purchased from Muromachi Yakuhin (Japan).

### RT-PCR and Southern hybridization

The procedures were the same as described elsewhere [31]. Rats were deeply anesthetized with ethyl ether and decapitated. The brains were then removed. Total RNAs including mRNA were isolated from adult rat tissues such as the hippocampus, hypothalamus, adrenal gland, kidney and liver, using a total RNA Purification Kit (Nippongene, Japan). The purified RNAs were quantified on the basis of the absorbance at 260/280 nm, and treated with RNase-free DNase to eliminate the possibility of genomic DNA contamination. The purified RNAs were reverse-transcribed, using a M-MLV Reverse Transcriptase (Promega, USA). The oligonucleotides for PCR amplification were designed as illustrated in Table S3. The PCR protocols comprised application of a 30 sec denaturation period at 95°C, a 2 sec annealing period at individual temperature for each enzyme, and a 30 sec extension at 72°C, for individual number of cycles for each enzyme (Table S3). For semiquantitative analysis, the RT-PCR products were separated on 2% agarose gels, stained with ethidium bromide, and analyzed with a fluorescence gel scanner (Atto, Japan) and Image J software, in comparison with standard curves obtained from PCR of diluted RT products (between 1/100 and 1/10,000 in dilution), from adrenal gland, kidney and liver.

To confirm the expression, Southern hybridization was performed. The amplified RT-PCR products of steroidogenic enzymes were directly cloned into TA-cloning vector (Promega, USA), and sequenced. The resulting sequence was identical to the reported cDNA sequences of these enzymes. These cloned products were used as the template of DNA probes for Southern hybridization. After transfer of the RT-PCR products from agarose gels to nylon membrane (Hybond N+, Amersham, USA), Southern hybridization was performed with ^32^P-labeled cDNA probes for these enzymes. The Southern hybridization signals were then measured using a BAS-1000 Image analyzer (Fuji film, Japan).

### *In situ* hybridization of hippocampal slices

Rats were deeply anesthetized with ethyl ether and decapitated. The brains were removed and the hippocampus was dissected. Preparation of adult hippocampal slices fixed with 4% paraformaldehyde, was performed essentially as described in elsewhere [8], [9], [12]. The postfixed brain tissue was frozen-sliced coronally at 15 µm thickness with a cryostat (Leica, Germany) and slices were immediately mounted on the slide glass at −18°C. Digoxigenin (DIG)-labeled sense and antisense cRNA probes were *in vitro* transcribed from PCR products of P450(11β1), P450(2D4) and P450(c21) by using T7 or Sp6 promoters inherent in pGEM-T-Easy vector. The hippocampal slices were treated with 10 µg/mL of Proteinase K (Wako, Japan) for 10–20 minutes, and then postfixed with fixative solution for 10 minutes. After acetylation with acetic anhydride and dehydration, the mRNA in hippocampal slices were hybridized with 0.5 µg/mL of DIG-labeled sense or antisense cRNA probes. In order to digest and wash out the excess cRNA probes, the slices were treated with RNase A (Wako, Japan) and stringent washes after hybridization. For P450(11β1) and P450(2D4), the slices were incubated with alkaline phosphatase-conjugated anti-DIG antibody (Roche Diagnosis, USA) (1/1000) for 30 min. After washing the hippocampal slices twice, target mRNAs were visualized by color development with 0.45 mg/mL of nitro blue tetrazolium chloride (NBT) and 0.175 mg/mL of 5-bromo-4-chloro-3-indoryl phosphate (BCIP) (Roche Diagnosis, USA) for 12 h. For P450(c21), the sections were incubated with horseradish peroxidase-conjugated anti-DIG antibody (Roche, USA) (1/1000) for 30 min. To amplify signals of *in situ* hybridization, the slides were incubated with dinitrophenyl labeled amplification reagent using the TSA Plus DNP (AP) System (Perkin Elmer, USA) and stained with NBT/BCIP for 12 h.

### Post-embedding immunogold method for electron microscopy

Rat hippocampal slices were prepared in essentially the same manner as described elsewhere [32], except that slicing was performed at 4°C using a vibratome instead of frozen slicing. Brains from five animals were used and from each brain, a single representative coronal section including the dorsal hippocampus was processed for ultrathin sectioning. Freeze substitution and low-temperature embedding of the specimens was performed as described previously [32]. Briefly, slices were plunged into liquid propane. The samples were immersed in uranyl acetate in anhydrous methanol (−90^°^C), then infiltrated with Lowicryl HM20 resin (Electron Microscopy Sciences, USA); polymerization was performed with ultraviolet light. Ultrathin sections were cut using a Reichert-Jung ultramicrotome. For immunolabeling, sections were incubated with primary antibody for 3β-HSD(type1) (1∶500) [33], P450(c21) (1∶2000) [34], P450(2D4) (1∶500) [16] or P450(11β) (1∶1000) [35] in the above diluent overnight, then incubated with secondary gold-tagged (10 nm) Fab fragment in Tris-buffered saline (TBS). Sections were counter-stained with 1 % uranyl acetate, and viewed on a JEOL 1200EX electron microscope (Japan). Images were captured using the CCD camera (Advanced Microscopy Techniques, USA). Controls omitting the primary antibody were performed and no immunogold labeling was observed. Controls of preadsorption incubating with purified antigens were also performed and no immunogold labeling was observed.

### Mass-spectrometric assay of steroids

Detailed procedures are described elsewhere [18] (Text S1).

#### Step 1) Purification of steroids from hippocampi with normal phase HPLC

The preparation of hippocampal homogenates from slices and the extraction of steroids were performed as described elsewhere [12]. ^3^H-steroids (20,000 cpm each) were added to homogenates as internal standards. The steroid extracts were applied to a C_18_ Amprep solid phase column (Amersham Biosciences, USA) to remove contaminating fats. Then the steroids were separated into fractions of CORT, DOC and PROG using a normal phase HPLC system (Jasco, Japan) with an elution solvent of hexane: isopropylalcohol: acetic acid  = 98∶2∶1. A silica gel column (Cosmosil 5SL, Nacalai Tesque, Japan) was used. By monitoring ^3^H-steroids, the recovery of CORT, DOC and PROG were 35±1%, 34±1%, and 42±1%, respectively, after extraction, C_18_ column treatment and normal phase HPLC separation. Plasma was prepared by centrifugation from trunk blood collected from decapitated rats [8].

#### Step 2) Determination of the concentration for CORT, DOC and PROG using LC-MS/MS

At first, 100 pg of isotope labeled steroids (^13^C_3_-PROG, DOC-d_8_ and CORT-d_8_) were added to steroid extracts prepared via *Step 1*). The LC-MS/MS system, which consisted of a reverse phase LC (Agilent 1100, Agilent Technologies, USA) coupled with an API 5000 triple-stage quadrupole mass spectrometer (Applied Biosystems, USA), was operated with electrospray ionization in the positive-ion mode.

The LC chromatographic separation was performed on a Cadenza CD-C_18_ column (3×150 mm, 3 µm, Imtakt Japan). Detailed conditions for column purification were described in Text S1 of Supporting Information. In the multiple reaction monitoring mode, the instrument monitored the m/z transition, from 347 to 121 for CORT, from 331 to 97 for DOC, and from 315 to 109 for PROG, respectively. Here, m and z represent the mass and charge of a steroid derivative, respectively.

To examine specificity of LC-MS/MS analysis, samples were spiked with steroid isotopes as internal standards. Though the m/z transitions were different between CORT (from m/z = 347 to 121) and CORT-d_8_ (from m/z = 355 to 125), their retention times were the same, because the affinity of CORT for LC-column is same as that for CORT-d_8_. In case of other steroids, there is also no difference in the retention time between steroids and their isotopes, though the m/z transitions were different. Isotope-labeled steroid derivatives were also used for internal standards in order to measure recovery of steroids. The recovery of CORT, DOC and PROG were determined as 89±8%, 75±4% and 71±6%, respectively, after purification and MS/MS detection. Total recovery during all the steps was determined via ^3^H- and isotope-labeled steroids in *Step 1*) and *Step 2*).

The limits of quantification for steroids were measured with blank samples, prepared alongside hippocampal samples through the whole extraction, fractionation and purification procedures. The limits of quantification for CORT, DOC and PROG were 2 pg, 1 pg, and 2 pg per 0.1 g of hippocampal tissue or 1 mL of plasma, respectively (Table S2).

From the calibration curve using standard steroids dissolved in blank samples, the linearity was observed between 2 pg and 4000 pg for CORT, between 1 pg and 1000 pg for DOC, and between 2 pg and 4000 pg for PROG, respectively (Fig S5).

Male Wistar rats were anesthetized with pentobarbital and placed in a stereotaxic Because the LC-MS/MS enabled to determine the exact level of CORT in the brain, we applied LC-MS/MS to observe the circadian rhythm of CORT level in the cerebrospinal fluid (CSF) in combination with the transverse microdialysis [36], [37]. Microdialysate was collected from the cisterna magna in freely moving male Wistar rats every hour (Fig. S6). Narishige apparatus (SR-5, Narishige, Japan) inserting a transverse dialysis tube placed at the cisterna magna under the guidance of a stainless steel wire attached in a horizontal position to a stereotaxic holder [37]. The rats were allowed roughly 1 week to recover from the surgery. Thereafter, the animals were moved to an acrylic test box with the transverse probe being perfused with a Ringer's solution (138 mM NaCl, 2.4 mM KCl, 1.2 mM CaCl_2_, [pH 7.0]) at 1 µL/min. Daily samples of CSF from the cisterna magna were automatically collected every hour in a small vial. The rats had free access to food placed on the floor of the test box and to water given from the lid of the box.

### Metabolism analysis of radioactive steroids using normal phase HPLC

Procedures were essentially the same as previously described in elsewhere [8], [12]. The hippocampus from one rat was sliced into 400 µm thickness with a vibratome and incubated with 5×10^6^ cpm of [^3^H]-steroids at 30°C for 5 h in 4 ml of physiological saline containing 1.2 mM Mg^2+^. The incubation medium was gassed with 95% O_2_ and 5% CO_2_ during the incubation in order to keep the activity of hippocampal neurons. After termination of the reaction, the slices were homogenized. A portion of the purified radioactive metabolites (total of 10^6^ cpm) was analyzed using an HPLC system. Procedures used for the analysis of steroid metabolites from hippocampal homogenates with normal phase HPLC were the same as described in *Step 1*) of Mass-spectrometric assay. Fraction radioactivity was measured using a liquid scintillation spectrometer LS6500 (Beckman, USA). The rate of steroid production was normalized as products (cpm) /g wet weight /5 h.

### Imaging and analysis of dendritic spine density and morphology

#### Slice preparation

Twelve weeks male rats were deeply anesthetized with ethyl ether and decapitated. Immediately after decapitation, the brain was removed from the skull and placed in ice-cold oxygenated (95% O_2_, 5% CO_2_) artificial cerebrospinal fluid (ACSF) containing (in mM): 124 NaCl, 5 KCl, 1.25 NaH_2_PO_4_, 2 MgSO_4_, 2 CaCl_2_, 22 NaHCO_3_, and 10 D-glucose (all from Wako); pH was set at 7.4. Hippocampal slices, 400 µm thick, were prepared with a vibratome (Dosaka, Japan). These slices were ‘freshly prepared’ slices without ACSF incubation. Slices were then incubated in oxygenated ACSF for 2 h (slice recovery processes) in order to obtain conventional ‘acute’ slices. These acute slices were then incubated at room temperature with CORT for 1 h. Then, slices were prefixed with 4% paraformaldehyde at 4°C for 2–4 h.

**Spine imaging and analysis** with confocal microscopy was performed as described previously [38]–[40].

#### Current injection of neurons by Lucifer Yellow

Neurons within slices were visualized by an injection of Lucifer Yellow under a Nikon E600FN microscope (Japan) equipped with a C2400–79H infrared camera (Hamamatsu Photonics, Japan) and with a 40× water immersion lens (Nikon). Dye was injected with a glass electrode filled with 5% Lucifer Yellow for 15 min, using Axopatch 200B (Axon Instruments, USA). Approximately five neurons within a 100–200 µm depth from the surface of a slice were injected (Duan H et al., 2002). After labeling, slices were fixed again with 4% paraformaldehyde at 4°C overnight.

#### Confocal laser microscopy and morphological analysis

Imaging was performed from sequential z-series scans with LSM5 PASCAL confocal microscope (Zeiss, Germany). For analysis of spines, three-dimensional images were constructed from approximately 40 sequential z-series sections of neurons scanned every 0.45 µm with a 63× water immersion lens, NA 1.2 (Zeiss). The excitation and emission wavelengths were 488 nm and 515 nm, respectively. The applied zoom factor (3.0) yielded 23 pixels per 1 µm. The z-axis resolution was approximately 0.71 µm. The confocal lateral resolution was approximately 0.26 µm. Our resolution limits were regarded as sufficient to allow the determination of the density of thorns or spines. Confocal images were then deconvoluted using AUTODEBLUR software (AutoQuant, USA). The density of spines as well as the head diameter was analyzed with Spiso-3D (automated software calculating geometrical parameters of spines) developed by Kawato's group of Bioinformatics Project [41]. Spiso-3D has an equivalent capacity with Neurolucida (MicroBrightField, USA), furthermore, Spiso-3D considerably reduces human errors and experimenter labor. We analyzed the secondary dendrites in the stratum radiatum, lying between 100 and 250 µm from the soma. The spine density was calculated from the number of spines having a total length of 50–80 µm. Spine shapes were classified into three categories as follows. (1) A small-head spine whose head diameter is 0.2–0.4 µm. (2) A middle-head spine whose head diameter is 0.4–0.5 µm. (3) A large-head spine whose head diameter is 0.5–1.0 µm. These three categories were useful to distinguish complex responses upon CORT application. Because the majority of spines (>95%) had a distinct head and neck, and stubby spines and filopodium did not contribute much to overall changes, we analyzed spines having a distinct head. While counting the spines in the reconstructed images, the position and verification of spines was aided by rotation of three-dimensional reconstructions and by observation of the images in consecutive single planes.

### Statistical analysis

Data were expressed as mean ± SEM. For Table 1, an unpaired, two-tailed *t*-test, under the assumption of unequal variances, was utilized to test the significance of observed differences between groups. Several numbers of independent experiments from different animals were used to determine the parameters of *t*-distribution for the test. For analysis of spinogenesis, we used Tukey–Kramer post hoc multiple comparisons test when one way ANOVA tests yielded P<0.05.

## Supporting Information

Text S1 **Supporting information for materials and methods, results, and discussions.**
(DOC)

Figure S1 **Pathways of corticosteroid synthesis in the hippocampus.** The abbreviated names of steroid (underline) and enzyme (italic) involved in each reaction are indicated.
(TIF)

Figure S2 **Gibbs free energy (ΔG) of P450c21 primer pairs used in the current study.** The vertical axis indicates ΔG of the primer and template DNA. The dotted line indicates the average of ΔG (ΔGav). We design these high sensitive primer pairs which have higher ΔG for the 3′-side primer than the 5′-side primer. The last five bases of the primers with the higher ΔG than ΔGav avoid the non-specific amplification (closed bar).
(TIF)

Figure S3 **Immunohistochemical staining for P450(11β1) in the hippocampus.** The coronal section of the whole hippocampus is used. P450(11β1) is expressed in pyramidal neurons in CA1-CA3 region and granule cells in DG. The expression of P450(11β1) in glial cells is weak. Scale bar, 800 µm.
(TIF)

Figure S4 **Western immunoblot analysis of P450c21 (A), P450(2D4) (B), P450(11β1) (C), and 3β-HSD (type 1) (D) in subcellular fractions of male rat hippocampus.** From left to right, postsynaptic membrane-rich fraction (Post), presynaptic membrane-rich fraction (Pre), postsynaptic density fraction (PSD), microsome (MS) and positive control. Adrenal gland (Ad) for (A) and (C), Liver (Li) for (B), and ovary (Ov) for (D) were used as positive control samples. The amount of protein applied to the gels is 20 µg for each hippocampal fraction, 0.5 µg for Ad, and 1 µg for ovary or liver.
(TIF)

Figure S5 **Calibration curves for LC-MS/MS using standard steroids dissolved in ethanol.** Horizontal (x) axis indicates the concentration of added standard steroid. Vertical (y) axis indicates the relative intensity obtained from the chromatogram. (A) Calibration curve for CORT. Linearity is observed between 2 pg/mL to 4000 pg/mL (in this figure only until 1000 pg/mL is shown). (B) Calibration curve for DOC. Linearity is observed between 1 pg/mL to 1000 pg/mL.
(TIF)

Figure S6 **Diurnal change of the concentration of CORT in the cerebrospinal fluid (CSF) from the cisterna magna in freely moving rats.** Rats with a microdialysis probe are maintained in the 12 hr light/12 hr dark cycle. Samples are collected every hour and 2 or 3 samples are combined and averaged. Data are expressed as mean ± SEM (n = 3). Three independent experiments with different animals were performed for each of these analyses, showing good reproducibility.
(TIF)

Table S1 **The accuracy of steroid determination for hippocampal tissue spiked with exogenous steroids.**
(DOC)

Table S2 **The intra- and inter-assay of accuracy and precision as well as the limit of quantification (LOQ) for each steroid.**
(DOC)

Table S3 **The sequence of primer oligonucleotides for PCR amplification.**
(DOC)
