# Supplementary material for: Endogenous Synthesis of Corticosteroids in the Hippocampus
Source: PLoS One. 2011 Jul 28;6(7):e21631. doi: 10.1371/journal.pone.0021631 (PMC3145636; doi:10.1371/journal.pone.0021631)
Supplement: Text S1 — Supporting information for materials and methods, results, and discussions. (DOC) [file pone.0021631.s001.doc]

# Supporting Information

# Materials and Methods

## Preparation of synaptic and other subcellular fractions

For Western blot analysis, whole hippocampal tissues were fractionated to synaptic and other subcellular fractions such as microsome and mitochondria. Fractionation was performed by a combination of centrifugations at 4 ^o^C [1,2]. Hippocampal tissues were homogenized in 0.32M sucrose in 1 mM NaHCO3, 1 mM MgCl2, 0.5 mM CaCl2 with protease inhibitors 0.5 mM PMSF and 0.1mM leupeptin, and centrifuged at 1,400 g for 10 min. The pellet was centrifuged again in the same solution. The pellet was used as the nuclear fraction (P1 fraction). Supernatant from the first centrifugation was subjected to centrifugation at 13,800 g for 10 min and separated to supernatant (S2) and pellet (P2). The retrieved pellet (P2 fraction) was further purified with sucrose density gradient centrifugation. The pellet was suspended in 0.32M sucrose in 1mM NaHCO3 and overlaid on 0.85/1.0/1.2 M sucrose step gradient. The sample was ultracentrifuged at 82,500 g for 120 min. The pellet under 1.2 M sucrose was used as the mitochondrial fraction. The synaptosomal fraction was collected from the interface between 1.0M and 1.2M sucrose. Purification of the ‘PSD (postsynaptic density) fraction’ was performed as follows. Synaptosomal fraction was treated with 1% Triton X-100 in 12mM Tris-HCl (pH 8.1), and Triton-insoluble pellet fraction were collected by centrifugation at 32,800 g for 20 min. The pellet was suspended in 0.32M sucrose in 1mM NaHCO3 and overlaid on 1.0/1.5/2.1 M sucrose step gradient. The sample was ultracentrifuged at 201,800 g for 120 min. Crude PSD fraction was collected from the interface between 1.5 M and 2.1 M sucrose, and then suspended in 150mM KCl with 1% TritonX-100. The suspension was ultracentrifuged for 20min at 201,800 g, and the pellet was collected as purified PSD fraction. To obtain the microsomal fraction (MS), the supernatant (S2) obtained from fractionation of P2 was centrifuged at 105,000 g for 60 min. The pellet and the supernatant was collected separately as the microsomal fraction and the cytoplasmic fraction, respectively.

## Western immunoblot analysis

Procedures were essentially same as that previously described [3]. Purified fractions prepared by centrifugation were suspended in 125 mM Tris-HCl buffer (pH 6.8) containing 5 mM 2-mercaptoethanol, 10% sucrose, 6% sodium dodecylsulfate (SDS) and 0.002% bromophenol blue. The fractions were subjected to electrophoresis using a 7.5% polyacrylamide gel for P450c21 and P450(11β1), and 10% for P450(2D4) and 3β-HSD (type 1), respectively. After transfer to polyvinylidene fluoride membranes (Immobilon-P; Millipore Co., Bedford, MA, USA), the blots were probed with anti-P450c21 (diluted to 1/2000, [4]), anti-P450(2D4) [5], anti- P450(11β1) [6], or anti-3β-HSD (type 1) antibodies [7] for 12–18 h at 4 ^o^C, and incubated with horseradish peroxidase (HRP)-conjugated goat anti-rabbit IgG (Cell Signaling). The protein bands were detected with enhanced chemiluminescence (ECL) plus western blotting detection reagents (Amersham, Piscataway, NJ, USA). To obtain high quality images of chemiluminescence from protein bands using ECL plus, we used LAS3000 Image Analyzer (Fuji Film) with a 16-bit wide dynamic range.

## Immunohistochemical staining of P450(11β1) in hippocampal slices

Immunohistochemical staining was performed essentially as described elsewhere [2,8]. Briefly, hippocampal slices were prepared from a rat or mouse deeply anesthetized with pentobarbital and perfused transcardially with PBS (0.1 M phosphate buffer and 0.14 M NaCl, pH 7.3), followed by fixative solution of 4% paraformaldehyde. The hippocampi were post-fixed, cryoprotected and frozen-sliced coronally at 20 μm thickness with a cryostat (Leica CM1510, Germany). Brains from several animals were used and from each brain, a single representative coronal section including the dorsal hippocampus was selected.

Staining for P450(11β1) was performed using the avidin-biotin peroxidase complex (ABC) technique. P450(11β1) was pre-treated with 5% BSA for 5 h to pre-absorb non-specific contaminated IgGs against BSA. After application of P450(11β1) antibodies (1/1000), the slices were incubated for 24 h at 4 ^o^C, in the presence of 0.5% Triton X-100 and 3% skim milk, with gentle shaking. Triton X-100 treatment was necessary to facilitate penetration of IgGs into cells in slices. Biotinylated anti-rabbit IgG (1/1000) in PBS was then applied, followed by a 30 min incubation with streptavidin-horseradish peroxidase complex (Vector Laboratories, Burlingame, CA, USA). Immunoreactive products were detected by immersing the slices in a detection solution (0.1 M Tris-HCl, pH 7.2, containing 0.05% diaminobenzidine, 0.1% H_2_O_2_ and 0.3% ammonium nickel sulfate). After dehydration and embedding in Entellan Neu (Merck), the immunoreactive cells in the slices were examined under a microscope, and digital images with a 2272 × 1704 pixel resolution were taken by a digital camera (COOLPIX4500, Nikon). For pre-absorption of P450(11β1) with antigen, excess amount of antigen was pre-incubated with P450(11β1) for 15 h at 4 ^o^C.

## Determination of the concentration for CORT, DOC and PROG using LC-MS/MS.

At first, 100 pg of isotope labeled steroids (^13^C_3_-PROG, DOC-d_8_ and CORT-d_8_) were added to steroid extracts prepared via *Step 1)* in order to calibrate the retention time for each steroid and examine the specificity of LC-MS/MS analysis described below*.* The LC-MS/MS system, which consisted of a reverse phase LC (Agilent 1100, Agilent Technologies, USA) coupled with an API 5000 triple-stage quadrupole mass spectrometer (Applied Biosystems, USA), was operated with electrospray ionization in the positive-ion mode.

The LC chromatographic separation was performed on a Cadenza CD-C_18_ column (3x150 mm, 3 μm, Imtakt Japan). The mobile phase composed of two solvents, solvent A (0.1% formic acid) and solvent B (acetonitrile:methanol 50:50), was delivered at flow rate of 0.4 ml/min. Total run time was 8 min. The initial conditions were held at the mixture of solvent A and B (50:50 v/v). After injection of 0.02 ml sample, this was followed by a linear gradient to 100 % solvent B for 4 min and then these conditions was maintained for 2 min. This system was returned to the initial proportion of the mixture of solvent A and B (50:50 v/v) within 0.05 min and maintained for the final 1.95 min of each run.

The ionization conditions of LC separated steroids were as follows: ion spray voltage, 5 kV; turbo gas temperature, 600 ºC; ion source gas 1 (nebulizer gas), 70 psi; ion source gas 2 (turbo gas), 70 psi; declustering potential, 70 V. Nitrogen was used as the collision gas in the Q2 collision cell.

In the multiple reaction monitoring mode, the instrument monitored the m/z transition, from 347 to 121 for CORT, from 331 to 97 for DOC, and from 315 to 109 for PROG, respectively. Here, m and z represent the mass and charge of a steroid derivative, respectively. In MS/MS procedures, for example, the mother ion (CORT, m/z = 347) is first selected using a 1st stage mass spectrometer. This CORT is then broken by collision with N_2_ gas, and the fragmented ion (m/z = 121) is selected using a 2nd stage mass spectrometer and detected.

To examine specificity of LC-MS/MS analysis, samples were spiked with steroid isotopes as internal standards. Though the m/z transitions were different between CORT (from m/z = 347 to 121) and CORT-d_8_ (from m/z = 355 to 125), their retention times were the same, because the affinity of CORT for LC-column is same as that for CORT-d_8_. In case of other steroids, there is also no difference in the retention time between steroids and their isotopes, though the m/z transitions were different. Isotope-labeled steroid derivatives were also used for internal standards in order to measure recovery of steroids. The recovery of CORT, DOC and PROG were determined as 89±8%, 75±4% and 71±6%, respectively, after purification and MS/MS detection. Total recovery during all the steps was determined via ^3^H- and isotope-labeled steroids in *Step 1)* and *Step 2)*.

The limits of quantification for steroids were measured with blank samples, prepared alongside hippocampal samples through the whole extraction, fractionation and purification procedures. The limits of quantification for CORT, DOC and PROG were 2 pg, 1 pg, and 2 pg per 0.1g of hippocampal tissue or 1 mL of plasma, respectively (Table S2). From the calibration curve using standard steroids dissolved in blank samples, the linearity was observed between 2 pg and 4000 pg for CORT, between 1 pg and 1000 pg for DOC, and between 2 pg and 4000 pg for PROG, respectively (Fig S5).

## Microdialysis of CSF

Male Wistar rats were anesthetized with pentobarbital and placed in a stereotaxic Narishige apparatus (SR-5, Narishige, Japan) inserting a transverse dialysis tube placed at the cisterna magna under the guidance of a stainless steel wire attached in a horizontal position to a stereotaxic holder [9]. The rats were allowed roughly 1 week to recover from the surgery. Thereafter, the animals were moved to an acrylic test box with the transverse probe being perfused with a Ringer’s solution (138 mM NaCl, 2.4 mM KCl, 1.2 mM CaCl_2_, [pH 7.0]) at 1 μL/min. Daily samples of cerebrospinal fluid (CSF) from the cisterna magna were automatically collected every hour in a small vial. The rats had free access to food placed on the floor of the test box and to water given from the lid of the box. Corticosteroid extraction/purification and determination were performed according to mass-spectrometric analysis as described in the text.

# Results and Discussion

## Primer design for successful detection of hippocampal P450(c21) mRNA

Generally, reduction of Gibbs free energy (ΔG) derived from the interaction between the primer and the target sequence of cDNA tends to increase the sensitivity of PCR analysis. On the other hand, the reduction of ΔG tends increase GC contents in the primer, resulting in the reduction of PCR specificity. Therefore, it is necessary to find the optimal primer sequence based on the balance of these two factors for constructing good primers. In the present study, primers were designed based on the following strategies:

(1) Calculating the averaged value of ΔG (ΔGav) for the interaction between all primer candidates and the target sequence in cDNA.

(2) Setting ΔG for a whole primer molecule below the ΔGav to obtain the good stability in primer-target interaction.

(3) Setting ΔG for 5 bases in 3’-side of the primer to be larger than ΔGav for improved specificity, because it is known that the specificity of primer-target recognition is mainly governed by 3’-side sequence of the primer.

As the result of (2) and (3), ΔG of the primer-target interaction in 5’-side is set to be below the ΔGav, which results not only in the improved stability in 5’-side interaction but also in the improved sensitivity of PCR detection caused by the improvement of polymerase recognition.

## Mass-spectrometric analysis of CORT in the hippocampus of stressed rat.

The average hippocampal volume was 0.14 mL (deduced from 0.14± 0.02 wet weight for 12 weeks old rat, n = 86). The average concentrations of CORT, DOC and PROG in the hippocampus of adrenal intact rats were calculated to be 128.1, 1.9 and 4.6 ng/g wet weight (i.e., 369.8, 5.9 and 14.6 nM), respectively. The relative concentrations of CORT, DOC and PROG were CORT >> PROG > DOC in this order. In plasma, the average concentrations of CORT, DOC and PROG were 510.3, 1.3 and 2.1 ng/mL (i.e., 1472.8, 3.8 and 6.8 nM), respectively. The concentration of DOC and PROG was higher in the hippocampus than in plasma, indicating the endogenous synthesis of DOC and PROG in the hippocampus.

These results imply that adrenal intact rats are not suitable for determination of the net hippocampus-synthesized CORT, due to the considerable elevation of hippocampal CORT by penetration of elevated plasma CORT induced by ether stress before decapitation. A part of plasma CORT (700-800 nM) above the buffering capacity of CBG (CORT binding globulin) (roughly 700 nM) probably penetrates into the hippocampus. To this end, we determined the net hippocampus-synthesized CORT by using ADX rats in order to eliminate the contribution of adrenal CORT.

## Diurnal change in CORT level in CSF

As an another important information of brain CORT, the CORT level in CSF was investigated in combination with the transverse microdialysis [9,10]. Microdialysate was collected from the cisterna magna in freely moving male Wistar rats every hour (n = 3). Diurnal change in CORT in the CSF was observed. The concentration of CORT in the CSF elevated roughly 5 fold higher in the dark period than that in the light period (Fig. S6), showing circadian rhythm. The observed CORT in CSF may be sum of brain-synthesized CORT and plasma CORT penetrated into CSF.

## Aldosterone (ALDO) production in the brain

Although DOC production from PROG has not been successfully demonstrated, ALDO production from DOC is shown [11-13]. Aldosterone synthesis is observed using DOC as substrate [12]. In the current study, P450(11β2) mRNA was expressed in the hippocampus (Fig. 1D), in accordance with the previous result [11]. De novo synthesis of ALDO in the brain is supported by the observation that some brain ALDO still presents in ADX rats (roughly 10 pg/g = 30 pM) [13]. However, PROG → DOC reaction in the brain as well as the brain expression of P450(c21) has not been demonstrated until the current study. In the current study, we demonstrated the expression and neuronal localization of P450(c21) as well as its activity of in the hippocampus. Taken together, these results suggest the existence of the complete ALDO synthesis pathway, that is,‘PREG → PROG → DOC → ALDO’in the hippocampus.

# Figure Legends

## Figure S1

Pathways of corticosteroid synthesis in the hippocampus. The abbreviated names of steroid (underline) and enzyme (italic) involved in each reaction are indicated.

## Figure S2

Gibbs free energy (ΔG) of P450c21 primer pairs used in the current study. The vertical axis indicates ΔG of the primer and template DNA. The dotted line indicates the average of ΔG (ΔGav). We design these high sensitive primer pairs which have higher ΔG for the 3’-side primer than the 5’-side primer. The last five bases of the primers with the higher ΔG than ΔGav avoid the non-specific amplification (closed bar).

## Figure S3

Immunohistochemical staining for P450(11β1) in the hippocampus. The coronal section of the whole hippocampus is used. P450(11β1) is expressed in pyramidal neurons in CA1-CA3 region and granule cells in DG. The expression of P450(11β1) in glial cells is weak. Scale bar, 800 μm

## Figure S4

Western immunoblot analysis of P450c21 (A), P450(2D4) (B), P450(11β1) (C), and 3β-HSD (type 1) (D) in subcellular fractions of male rat hippocampus. From left to right, postsynaptic membrane-rich fraction (Post), presynaptic membrane-rich fraction (Pre), postsynaptic density fraction (PSD), microsome (MS) and positive control. Adrenal gland (Ad) for (A) and (C), Liver (Li) for (B), and ovary (Ov) for (D) were used as positive control samples. The amount of protein applied to the gels is 20 μg for each hippocampal fraction, 0.5 μg for Ad, and 1 μg for ovary or liver.

## Figure S5

Calibration curves for LC-MS/MS using standard steroids dissolved in ethanol. Horizontal (x) axis indicates the concentration of added standard steroid. Vertical (y) axis indicates the relative intensity obtained from the chromatogram. (A) Calibration curve for CORT. Linearity is observed between 2 pg/mL to 4000 pg/mL (in this figure only until 1000 pg/mL is shown). (B) Calibration curve for DOC. Linearity is observed between 1 pg/mL to 1000 pg/mL.

## Figure S6

Diurnal change of the concentration of CORT in the cerebrospinal fluid (CSF) from the cisterna magna in freely moving rats. Rats with a microdialysis probe are maintained in the 12hr light/12 hr dark cycle. Samples are collected every hour and 2 or 3 samples are combined and averaged. Data are expressed as mean ± SEM (n = 3). Three independent experiments with different animals were performed for each of these analyses, showing good reproducibility.

## Table S1

The accuracy of steroid determination for hippocampal tissue spiked with exogenous steroids.

## Table S2

The intra- and inter-assay of accuracy and precision as well as the limit of quantification (LOQ) for each steroid.

## Table S3

The sequence of primer oligonucleotides for PCR amplification.

References

1. Cohen RS, Blomberg F, Berzins K, Siekevitz P (1977) The structure of postsynaptic densities isolated from dog cerebral cortex. I. Overall morphology and protein composition. J Cell Biol 74: 181-203.

2. Kawato S, Hojo Y, Kimoto T (2002) Histological and metabolism analysis of P450 expression in the brain. Methods Enzymol 357: 241-249.

3. Mukai H, Tsurugizawa T, Murakami G, Kominami S, Ishii H, et al. (2007) Rapid modulation of long-term depression and spinogenesis via synaptic estrogen receptors in hippocampal principal neurons. J Neurochem 100: 950-967.

4. Shinzawa K, Ishibashi S, Murakoshi M, Watanabe K, Kominami S, et al. (1988) Relationship between zonal distribution of microsomal cytochrome P-450s (P-450(17)alpha,lyase and P-450C21) and steroidogenic activities in guinea-pig adrenal cortex. J Endocrinol 119: 191-200.

5. Kishimoto W, Hiroi T, Shiraishi M, Osada M, Imaoka S, et al. (2004) Cytochrome P450 2D catalyze steroid 21-hydroxylation in the brain. Endocrinology 145: 699-705.

6. Ogishima T, Suzuki H, Hata J, Mitani F, Ishimura Y (1992) Zone-specific expression of aldosterone synthase cytochrome P-450 and cytochrome P-45011 beta in rat adrenal cortex: histochemical basis for the functional zonation. Endocrinology 130: 2971-2977.

7. Doody KM, Carr BR, Rainey WE, Byrd W, Murry BA, et al. (1990) 3 beta-hydroxysteroid dehydrogenase/isomerase in the fetal zone and neocortex of the human fetal adrenal gland. Endocrinology 126: 2487-2492.

8. Kimoto T, Tsurugizawa T, Ohta Y, Makino J, Tamura H, et al. (2001) Neurosteroid synthesis by cytochrome p450-containing systems localized in the rat brain hippocampal neurons: N-methyl-D-aspartate and calcium-dependent synthesis. Endocrinology 142: 3578-3589.

9. Nakahara D, Nakamura M, Iigo M, Okamura H (2003) Bimodal circadian secretion of melatonin from the pineal gland in a living CBA mouse. Proc Natl Acad Sci U S A 100: 9584-9589.

10. Ishida A, Mutoh T, Ueyama T, Bando H, Masubuchi S, et al. (2005) Light activates the adrenal gland: timing of gene expression and glucocorticoid release. Cell Metab 2: 297-307.

11. MacKenzie SM, Clark CJ, Fraser R, Gomez-Sanchez CE, Connell JM, et al. (2000) Expression of 11beta-hydroxylase and aldosterone synthase genes in the rat brain. J Mol Endocrinol 24: 321-328.

12. Gomez-Sanchez CE, Zhou MY, Cozza EN, Morita H, Foecking MF, et al. (1997) Aldosterone biosynthesis in the rat brain. Endocrinology 138: 3369-3373.

13. Gomez-Sanchez EP, Ahmad N, Romero DG, Gomez-Sanchez CE (2005) Is aldosterone synthesized within the rat brain? Am J Physiol Endocrinol Metab 288: E342-346.
