# Supplementary figures and images for: Endogenous Synthesis of Corticosteroids in the Hippocampus
Source: PLoS One. 2011 Jul 28;6(7):e21631. doi: 10.1371/journal.pone.0021631 (PMC3145636; doi:10.1371/journal.pone.0021631)

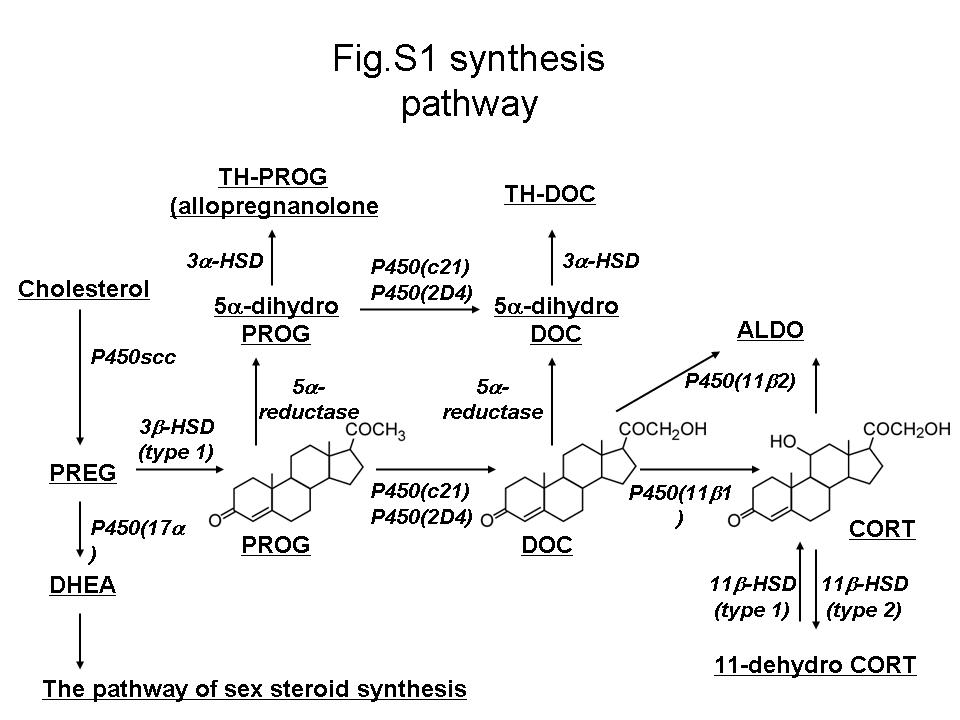

Supplement: Figure S1 — Pathways of corticosteroid synthesis in the hippocampus. The abbreviated names of steroid (underline) and enzyme (italic) involved in each reaction are indicated. (TIF) [file pone.0021631.s002.tif]

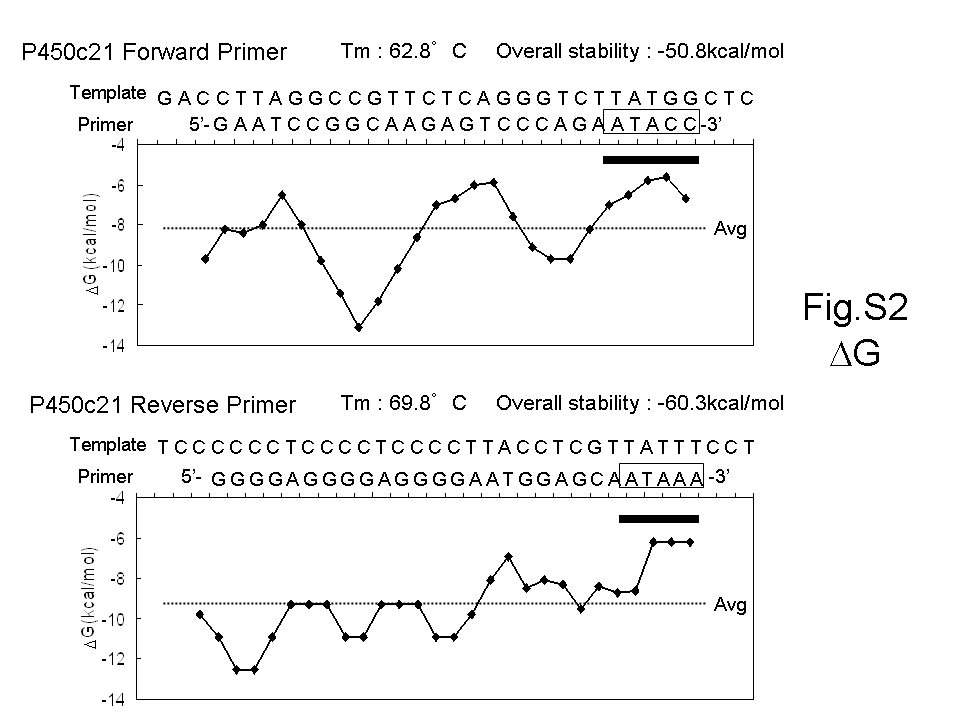

Supplement: Figure S2 — Gibbs free energy (ΔG) of P450c21 primer pairs used in the current study. The vertical axis indicates ΔG of the primer and template DNA. The dotted line indicates the average of ΔG (ΔGav). We design these high sensitive primer pairs which have higher ΔG for the 3′-side primer than the 5′-side primer. The last five bases of the primers with the higher ΔG than ΔGav avoid the non-specific amplification (closed bar). (TIF) [file pone.0021631.s003.tif]

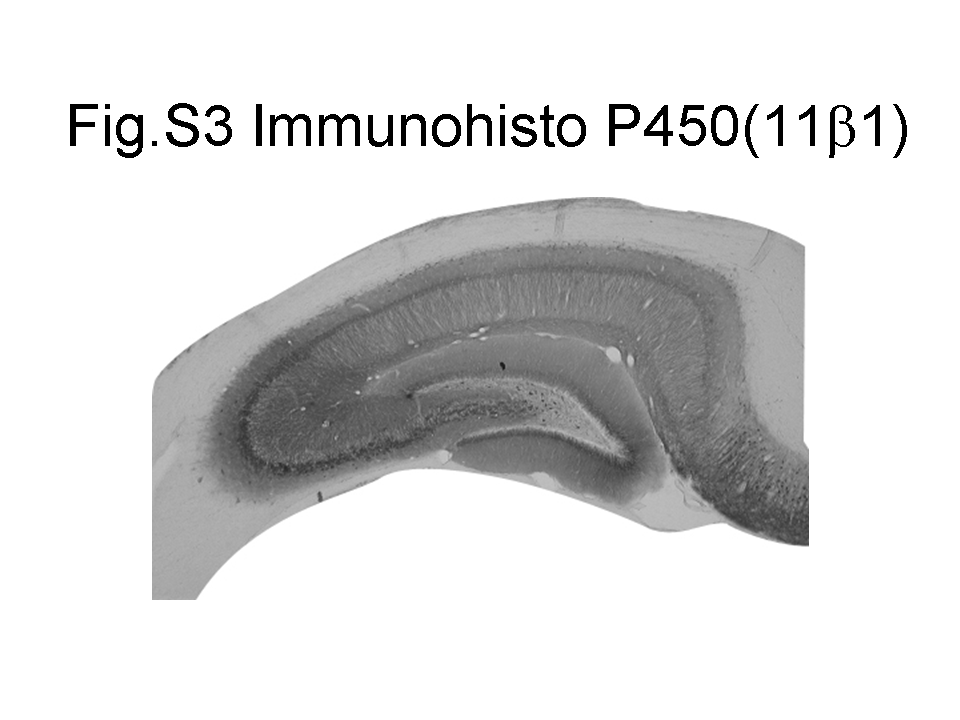

Supplement: Figure S3 — Immunohistochemical staining for P450(11β1) in the hippocampus. The coronal section of the whole hippocampus is used. P450(11β1) is expressed in pyramidal neurons in CA1-CA3 region and granule cells in DG. The expression of P450(11β1) in glial cells is weak. Scale bar, 800 µm. (TIF) [file pone.0021631.s004.tif]

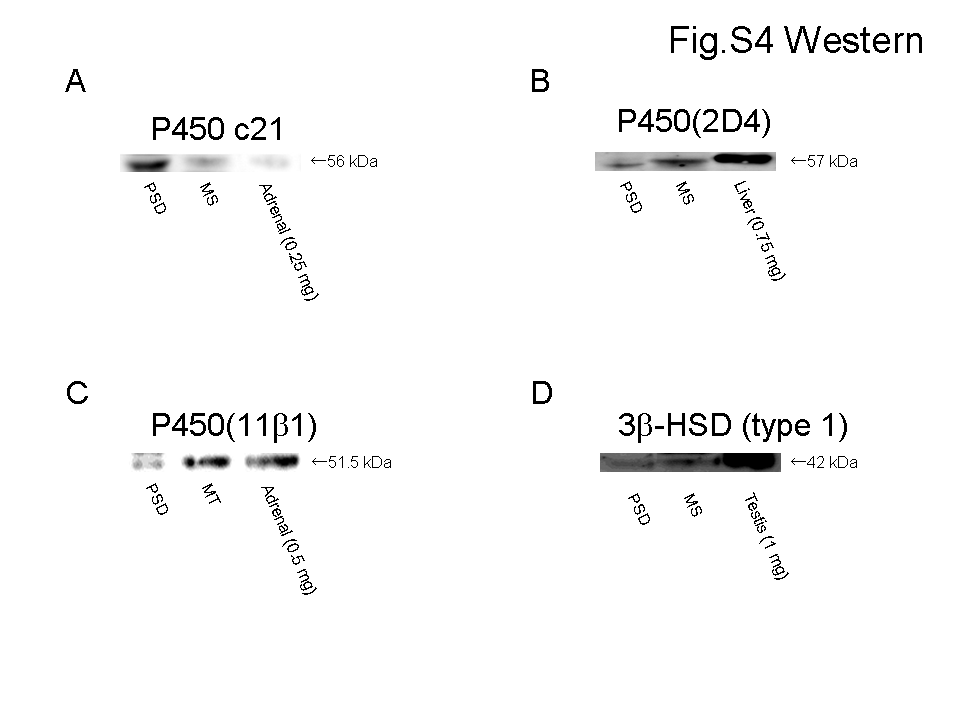

Supplement: Figure S4 — Western immunoblot analysis of P450c21 (A), P450(2D4) (B), P450(11β1) (C), and 3β-HSD (type 1) (D) in subcellular fractions of male rat hippocampus. From left to right, postsynaptic membrane-rich fraction (Post), presynaptic membrane-rich fraction (Pre), postsynaptic density fraction (PSD), microsome (MS) and positive control. Adrenal gland (Ad) for (A) and (C), Liver (Li) for (B), and ovary (Ov) for (D) were used as positive control samples. The amount of protein applied to the gels is 20 µg for each hippocampal fraction, 0.5 µg for Ad, and 1 µg for ovary or liver. (TIF) [file pone.0021631.s005.tif]

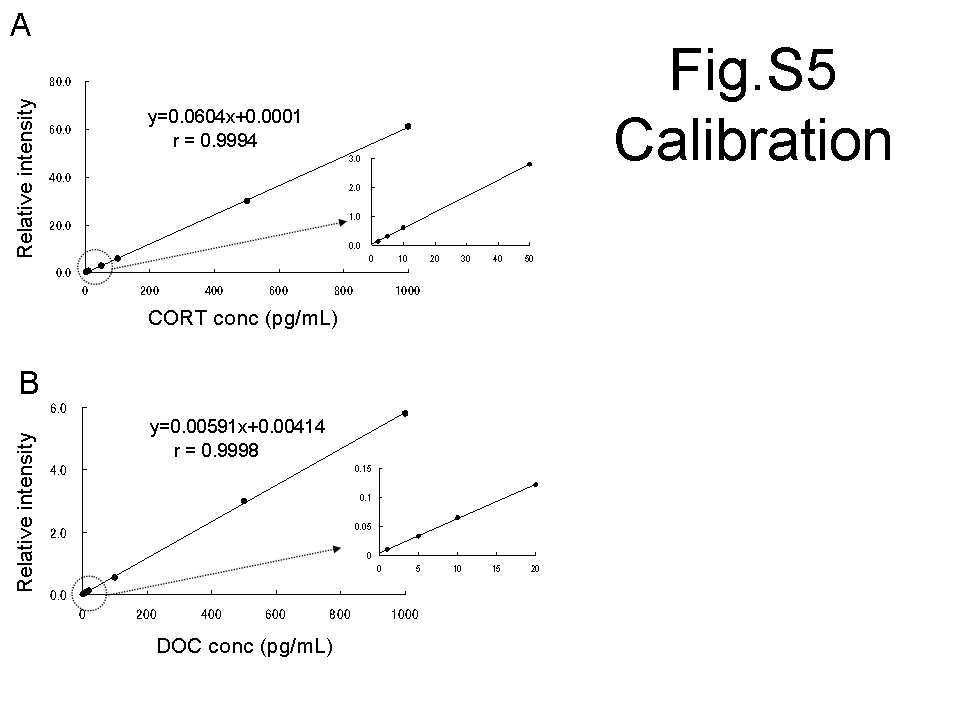

Supplement: Figure S5 — Calibration curves for LC-MS/MS using standard steroids dissolved in ethanol. Horizontal (x) axis indicates the concentration of added standard steroid. Vertical (y) axis indicates the relative intensity obtained from the chromatogram. (A) Calibration curve for CORT. Linearity is observed between 2 pg/mL to 4000 pg/mL (in this figure only until 1000 pg/mL is shown). (B) Calibration curve for DOC. Linearity is observed between 1 pg/mL to 1000 pg/mL. (TIF) [file pone.0021631.s006.tif]

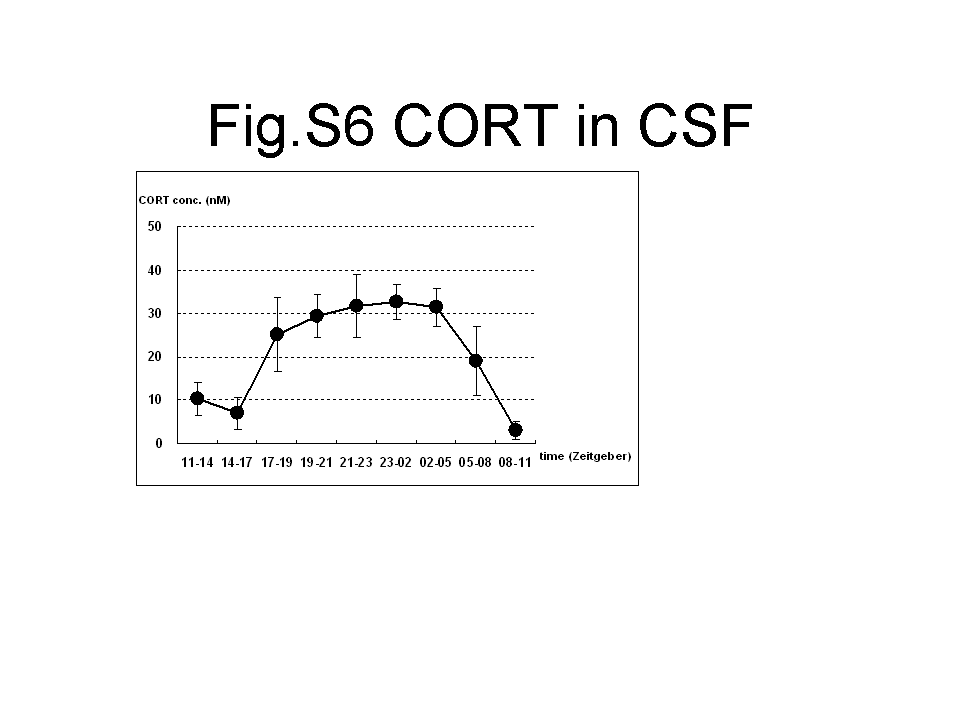

Supplement: Figure S6 — Diurnal change of the concentration of CORT in the cerebrospinal fluid (CSF) from the cisterna magna in freely moving rats. Rats with a microdialysis probe are maintained in the 12 hr light/12 hr dark cycle. Samples are collected every hour and 2 or 3 samples are combined and averaged. Data are expressed as mean ± SEM (n = 3). Three independent experiments with different animals were performed for each of these analyses, showing good reproducibility. (TIF) [file pone.0021631.s007.tif]
