# Supplementary material for: Endogenous Synthesis of Corticosteroids in the Hippocampus
Source: PLoS One. 2011 Jul 28;6(7):e21631. doi: 10.1371/journal.pone.0021631 (PMC3145636; doi:10.1371/journal.pone.0021631)
Supplement: Table S1 — The accuracy of steroid determination for hippocampal tissue spiked with exogenous steroids. (DOC) [file pone.0021631.s008.doc]

# Table S1

The accuracy of steroid determination for hippocampal tissue spiked with exogenous steroids.

|  | Steroid | Added (ng/g) | Found (ng/g) | Accuracy (%)^a^ |
| --- | --- | --- | --- | --- |
| Hippocampus | CORT | 0.0 | 362.421 | - |
|  |  | 0.5 | 362.955 (0.534)^b^ | 106.8 |
|  |  | 1.0 | 363.477 (1.056)^b^ | 105.6 |
|  | DOC | 0.0 | 1.916 | - |
|  |  | 0.5 | 2.302 (0.486)^b^ | 97.2 |
|  |  | 1.0 | 3.004 (1.088)^b^ | 108.8 |
|  | PROG | 0.0 | 4.911 | - |
|  |  | 0.5 | 5.384 (0.473)^b^ | 94.6 |
|  |  | 1.0 | 5.895 (0.984)^b^ | 98.4 |

^a^ Accuracy was expressed as a recovery rate (%) of the measured amount of steroid to the added amount of steroid.

^b^ Value in parentheses was obtained by subtraction of the endogenous amount of steroid from the value obtained for each spiked steroid.
