# Supplementary material for: Endogenous Synthesis of Corticosteroids in the Hippocampus
Source: PLoS One. 2011 Jul 28;6(7):e21631. doi: 10.1371/journal.pone.0021631 (PMC3145636; doi:10.1371/journal.pone.0021631)
Supplement: Table S2 — The intra- and inter-assay of accuracy and precision as well as the limit of quantification (LOQ) for each steroid. (DOC) [file pone.0021631.s009.doc]

# Table S2

The intra- and inter-assay of accuracy and precision as well as the limit of quantification (LOQ) for each steroid.

|  |  |  | Intraassay (n^a^ = 5) | | Interassay (n^a^ = 3) | |  |
| --- | --- | --- | --- | --- | --- | --- | --- |
|  | m/z^b^ transition | Spike  (pg) | Accuracy  (%) | RSD ^c^  (%) | Accuracy  (%) | RSD  (%) | LOQ ^d^  (pg/0.1g) |
| CORT | from 347  to 121 | 4 | 92.2 | 9.6 | 107.3 | 10.9 | 2 |
|  |  | 10 | 94.7 | 7.7 | 96.8 | 7.2 |  |
|  |  | 100 | 103.4 | 3.2 | 102.2 | 4.9 |  |
| DOC | from 331  to 108 | 1 | 88.8 | 10.2 | 91.6 | 9.2 | 1 |
|  |  | 10 | 95.7 | 8.2 | 97.6 | 8.3 |  |
|  |  | 100 | 98.2 | 1.8 | 101.7 | 5.5 |  |
| PROG | from 315  to 109 | 2 | 107.1 | 5.2 | 104.4 | 5.1 | 2 |
|  |  | 10 | 105.6 | 3.7 | 95.9 | 4.1 |  |
|  |  | 100 | 102.3 | 1.8 | 96.1 | 1.2 |  |

Blank samples, prepared alongside hippocampal samples through the whole extraction and purification procedures, were spiked with CORT or other steroids at 1, 2, 4, 5,10, 20 and 100 pg, and contents were determined by LC-MS/MS. Accuracy was expressed as a percentage of an analytical recovery rate of measured steroid content against spike amount.

^a^For each condition, intra- and interassay were performed five and three times, respectively.

^b^m and z represent the mass and charge of a steroid derivative, respectively.

^c^relative standard deviation.

^d^LOQ is expressed as pg/0.1 g. Because the average weight of one whole adult hippocampus (0.14 g) was close to 0.1 g, these LOQ values indicate the limit of quantification of steroids from nearly one hippocampus.
