# Supplementary material for: Endogenous Synthesis of Corticosteroids in the Hippocampus
Source: PLoS One. 2011 Jul 28;6(7):e21631. doi: 10.1371/journal.pone.0021631 (PMC3145636; doi:10.1371/journal.pone.0021631)
Supplement: Table S3 — The sequence of primer oligonucleotides for PCR amplification. (DOC) [file pone.0021631.s010.doc]

# Table S3

The sequence of primer oligonucleotides for PCR amplification.

| target mRNA |  | sequence | Ta(°C) | PCR cycles |
| --- | --- | --- | --- | --- |
| P450(c21) primer1 | forward | GAATCCGGCAAGAGTCCCAGAATACC | 64 | 38 |
|  | reverse | GAGGGGAGGGGAATGGAGCAATAAAG |  |  |
| P450(c21) primer2 | forward | GACCACGGCTGCCACGCTCTC | 64 | 38 |
|  | reverse | CGCCTTGGATGTTGGGGATGATG |  |  |
| P450(2D4) | forward | GACCAGTCGGGCTTTGGACCAC | 62 | 24 |
|  | reverse | CGAAGGCCTTCTTTCCAGAG |  |  |
| P450(11β1) | forward | AGACTGGCGCTTCAACCGACTG | 65 | 32 |
|  | reverse | ACATGGCCTTCTGGGGATTAGCAACG |  |  |
| P450(11β2) | forward | TGGCAGCACTAATAACTCAGG | 63 | 34 |
|  | reverse | TAGGGCCCCAGATGTAAGGT |  |  |
| 11β-HSD1 | forward | ATTGCCGGCACTATGGAAGAC | 55 | 24 |
|  | reverse | CTATGAAGCCGAGGACACAGAGA |  |  |
| 11β-HSD2 | forward | GCCCTGCTGGCCGCTCTCG | 65 | 34 |
|  | reverse | ACCAGGGCCATTCAAATCCAACACAG |  |  |
